# Superhydrophobic porous networks for enhanced droplet shedding

**DOI:** 10.1038/srep33817

**Published:** 2016-09-20

**Authors:** Yahua Liu, Zuankai Wang

**Affiliations:** 1 Key Laboratory for Precision and Non-traditional Machining Technology of Ministry of Education, Dalian University of Technology, Dalian 116024, China; 2 Department of Mechanical and Biomedical Engineering, City University of Hong Kong, Hong Kong, China; 3 Shenzhen Research Institute of City University of Hong Kong, Shenzhen 518057, China

## Abstract

Recent research has shown that the use of submillimeter-scale tapered post arrays could generate the so-called pancake bouncing, which is characterized by the fast shedding of impinging drops from the surface in a pancake shape without undergoing the retraction stage as observed on conventional superhydrophobic surfaces. Despite this exciting discovery, the fabrication of this unique superhydrophobic surface with tapered post arrays involves complex processes, hindering its wide applications in practical sectors. Here, we report on the facile strategy to prepare a new hierarchical multilayered superhydrophobic surface directly from commercially available porous matrix that allows for efficient drop shedding. Further study shows that the enhanced drop mobility observed on such a surface is attributed to the synergistic cooperation of hierarchical structures endowing an adequate energy storage and effective energy release. The facile fabrication of superhydrophobic surface with enhanced drop mobility may find many practical applications including anti-icing, dropwise condensation and self-cleaning.

Superhydrophobic surfaces, which have a water contact angle greater than 150°, have attracted increasing attention within the scientific community because of their numerous applications, including self-cleaning^1^, anti-corrosion^2^, anti-icing^3^ and drag reduction^4^. It is well accepted that superhydrophobicity is conferred by both hierarchical roughness and hydrophobic coating. Recently, superhydrophobic surfaces feathering different morphologies have been fabricated to achieve various applications^5^,^6^,^7^,^8^,^9^,^10^,^11^,^12^,^13^,^14^,^15^,^16^,^17^. Despite over a decade of intensive research, these surfaces are still plagued with problems that restrict their practical applications. For instance, the contact time between a impacting drop and a solid surface is constant because it is independent of the impact velocity for the classical dynamic process including spreading, retracting, and bouncing off^18^. Moreover, the entire dynamic process controls the extent to which mass, momentum, and energy are exchanged between a drop and a surface, leading to the optimal minimization of contact time. Recently, Bird *et al.* implemented a strategy that they induced droplet break-up by creating macroscale patterns on a superhydrophobic surface. When the drop impinges on the macroscale ridges, it splits into smaller ones, which rebounds from the surface in a shorter time as compared with the non-splitting condition^6^. The contact time was also shortened when a drop impacts on the ridge with precisely controlled impact velocities in which the drop keeps an integral shape^19^. More recently, Liu *et al.* observed a pancake bouncing on the specially designed millimeter-scale post arrays, and the contact time was reduced by ~80%^7^,^20^,^21^. However, the fabrication of this unique superhydrophobic surface with tapered posts involves the mechanical wire-cutting and chemical etching approaches, which is not suitable for practical applications owing to its high cost and complex processing^21^.

Here we report on the facile fabrication of a kind of hierarchical multilayered superhydrophobic (HMS) surface. The HMS surface is based on porous copper foam that is easy to be scaled up for practical applications. We show that an impacting liquid drop can directly rebound off the HMS surface in a pancake shape without undergoing the lateral drop retraction. The so-called pancake bouncing is characterized by about 60% reduction in contact time compared with that on conventional superhydrophobic surfaces. Using combined experimental and analytical analyses, we find that this transient phenomenon is endowed by the intricate interplay between the porous structure which permits the storage of adequate surface energy in a localized region and the effective release of the stored energy due to a matching between the lateral and vertical liquid motion. We believe that the easy fabrication of the bulk superhydrophobic material for fast drop shedding may find promising applications including self-cleaning, anti-icing, and dropwise condensation^1^,^22^,^23^,^24^,^25^.

## Experimental Section

### Surface fabrication

The solid substrate used in our experiments is hierarchical multilayered copper foam which is commercially available from Shanghai Zhongwei New Materials Co., Ltd. The as-received copper foam with density 0.45 g cm^−3^, porosity 94% and thickness 0.16 cm, was cut into 2.5 × 2.5 cm^2^ slides. The copper foam slides were ultrasonically cleaned in ethanol and deionized water for 10 min respectively and dried in nitrogen stream, followed by immersing in a freshly mixed aqueous solution of 2.5 mol L^−1^ sodium hydroxide and 0.1 mol L^−1^ ammonium persulphate at room temperature for ∼60 min, after which they were fully rinsed with deionized water and dried again in nitrogen stream. All the surfaces were modified by silanization immersing in 1 mM n-hexane solution of trichloro-(1H, 1H, 2H, 2H)-perfluorooctylsilane for ∼60 min, followed by heat treatment at ∼150 °C in air for 1 h.

### Characterization

**Figure 1:**
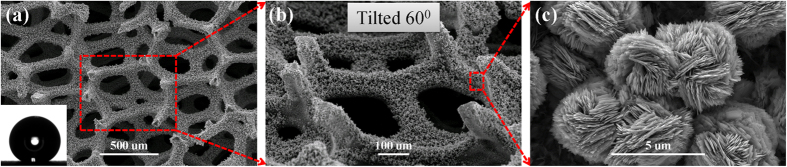
Scanning electron microscope (SEM) images of the hierarchical multilayered superhydrophobic (HMS) surface. (**a**) SEM images with low amplification. The left inset shows the optical image of a water drop deposited on the HMS substrate. (**b**) 60° side-view of the HMS substrate. All the surfaces are uniformly coated by nanostructured flowers with an intrinsic contact angle more than 160°. (**c**) Nanoflowers are coated on the surface.

**Figure 2:**
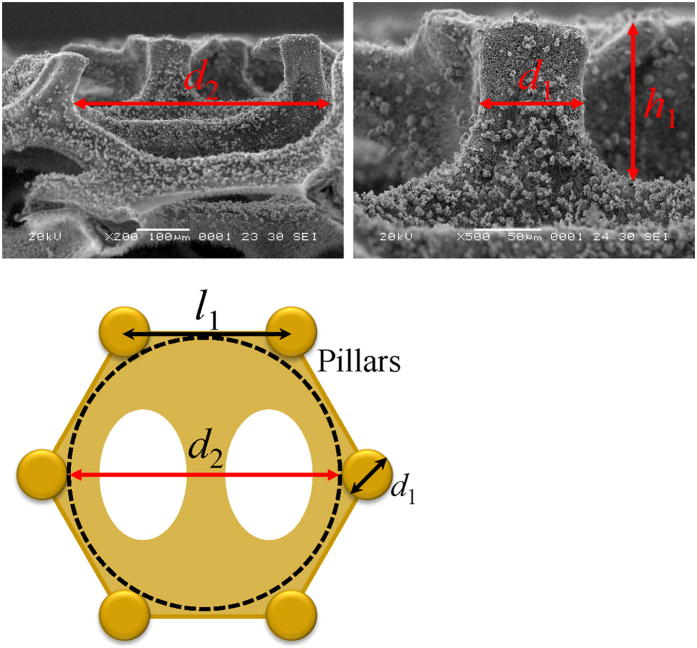
SEM images and schematic illustrations showing the structure of the surface. The pillar diameter (*d*_1_), height (*h*_1_), and pillar-to-pillar spacing (*l*_1_) of the first layer are 80, 200, and 260 μm, respectively. The characteristic pore diameter (*d*_2_) in the porous media is ~450 μm.

The micro/nano structures of HMS surface were characterized by a field-emission scanning electron microscope (SEM, Quanta 250 FEG). As shown in the SEM images in Fig. 1a–c, the first layer of the substrate are micropillar arrays with a hexagonal lattice arrangement, standing on multilayered porous media. The pillar diameter (*d*_1_), height (*h*_1_), and pillar-to-pillar spacing (*l*_1_) of the first layer are 80, 200, and 260 μm, respectively. The characteristic pore diameter (*d*_2_) in the porous media is ~450 μm. The resulting micro-structures are shown in Fig. 2. All the surfaces are uniformly deposited by nanostructured flowers with an average diameter of 3 μm (Fig. 1c). The intrinsic contact angle on the surface is over 160°. Note that, the static contact angle on the HMS surface was measured from sessile water drops with a Drop Shape Analyzer (DSA-100S). A deionized water drop of 4.2 μl, was deposited at a volume rate of 0.5 μl s^−1^, at room temperature with 60% relative humidity. At least five individual measurements were performed on the surface.

## Results and Discussion

### Drop impact experiments

We conducted water drop impact experiments on the HMS substrate under different velocities in ambient environment, at room temperature with 60% relative humidity. Briefly, a Milli-Q water drop of ~13 ml (with drop radius *r*_0_ ≈ 1.45 mm) was released from a fine needle equipped with a syringe pump (KD Scientific Inc.). The impact dynamics of drop was recorded by a high-speed camera (Fastcam SA4, Photron limited) at a frame rate of 10,000 fps with a shutter speed 1/93,000 s. The drop impacting dynamics was measured using ImageJ software (version 1.46, National Institutes of Health, Bethesda, MD).

**Figure 3:**
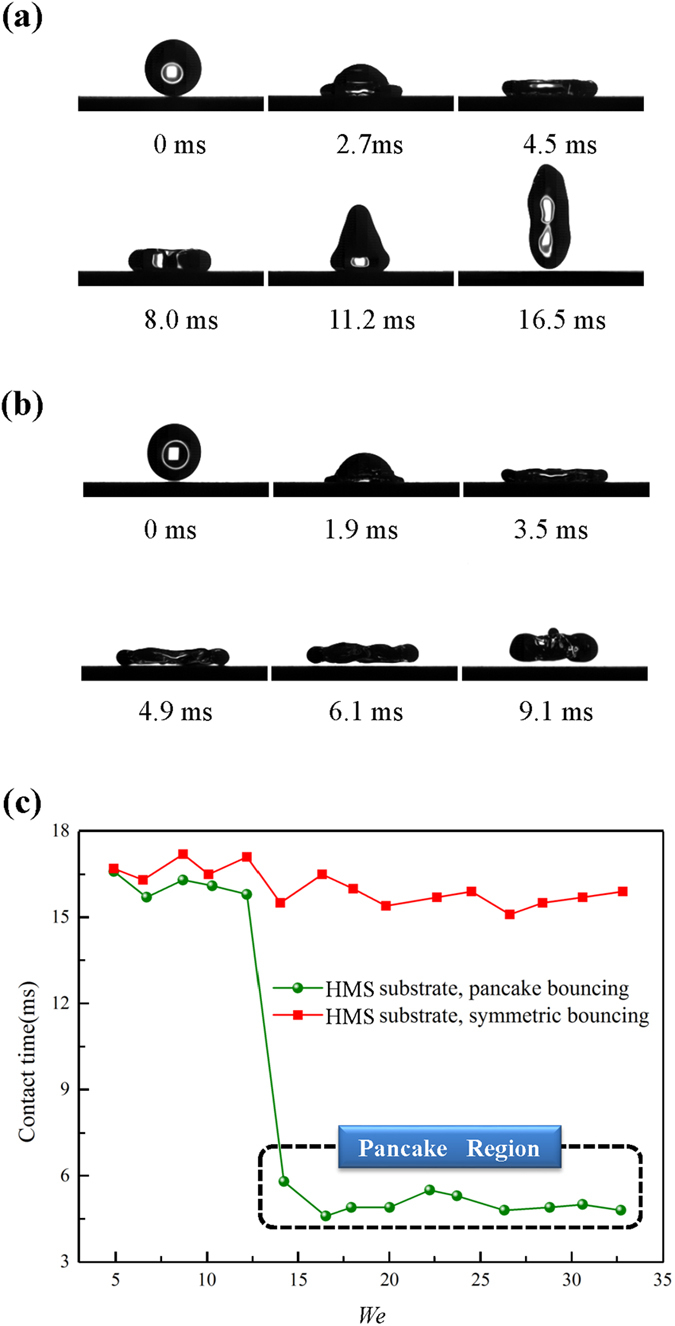
Selected snapshots of drop impact and the comparision of the contact time. (**a**) Selected snapshots captured by the high speed camera showing the drop impact on the HMS substrate under *We* = 8.7. (**b**) High speed camera snapshots showing the drop impact on the HMS substrate under *We* = 19.6. (**c**) Comparision of the contact time between the symmetric and pancake bouncing, indicating that the pancake bouncing has a much shortened contact time.

At a low impinging velocity, the drop exhibits a complete rebound, as reported on conventional superhydrophobic surfaces^18^. Figure 3a illustrates the selected high speed camera snapshots of drop impacting on the HMS substrate with an impinging velocity *v*_0_ of 0.65 m s^−1^, which yields a 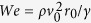 of 8.7 with 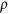 and *γ* being the density and surface tension of water drop, respectively. Note that in such a complete rebound, the drop always maintains a direct contact with the underlying substrate during the spreading and retraction stages (symmetric bouncing, from 0 to 16.5 ms). Owing to the elasticity of the impact, drop impacting on the superhydrophobic substrate can be treated as an oscillation with the contact time (=16.5 ms) independent of the drop impinging velocity^18^,^26^.

Interestingly, when the impinging velocity *v*_0_ is above a threshold 0.84 m s^−1^, corresponding to a *We* of 14.2, the drop directly rebounds off the surface in a pancake shape without undergoing the lateral drop retraction. As a characteristic example, Fig. 3b shows the drop impact dynamics over time on the HMS substrate with an impinging velocity *v*_0_ of 0.97 m s^−1^, or a *We* of 19.6. Impact experiments at higher velocity (less than 1.26 m s^−1^) confirmed the same phenomenon. Moreover, in the case of this unique bouncing, the contact time of the drop with the solid surface is nearly two-time less than that for the elastic bouncing (as shown in Fig. 3c). This bouncing phenomenon is in stark contrast to the drop bouncing on the HMS substrate at a low *We* or on conventional superhydrophobic substrates. Additionally, at higher velocity, we observed some daughter drops were trapped in the HMS substrate and the pancake bouncing phenomenon disappeared, indicating that the pancake bouncing phenomenon occurs with a particular preference on the impinging velocity.

### Characterization of drop impact dynamics

**Figure 4:**
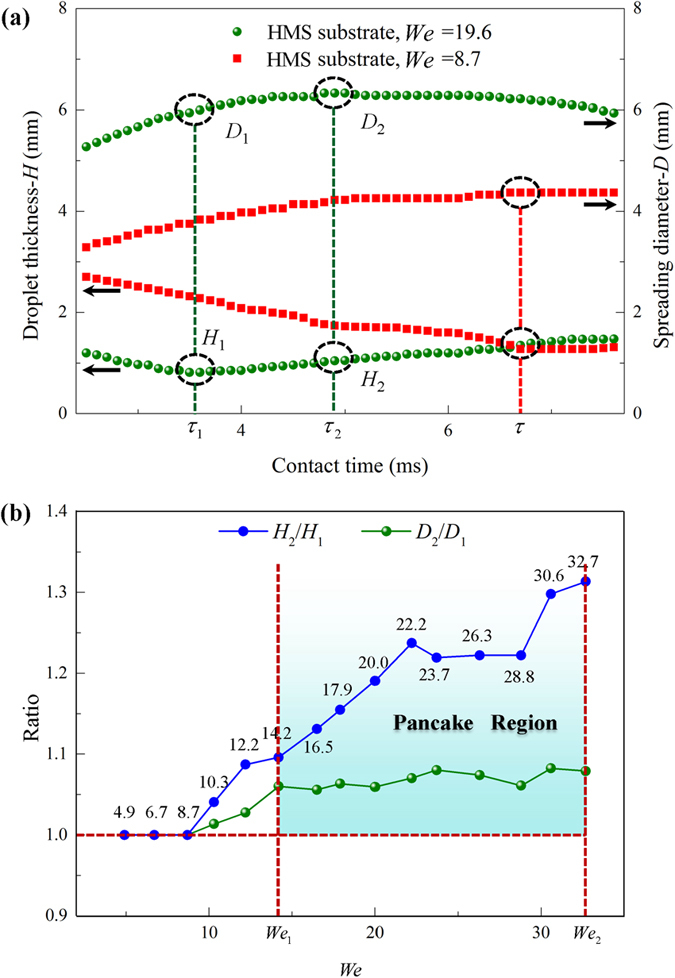
Temporal evolution of drop impact and rebound. (**a**) Temporal evolution of the spreading diameter *D*(*t*) and the thickness *H*(*t*) of the drop during its impact on the HMS substrate under *We* of 8.7 (symmetric bouncing) and 19.6 (pancake bouncing), respectively. Notably, the maximum spreading diameter and maximum thickness is aligned in the case of symmetric bouncing whereas they are seperated in the pancake bouncing. (**b**) The ratios *D*_2_/*D*_1_ and *H*_2_/*H*_1_ are plotted versus the *We*. These ratios are always larger than the unity in the pancake bouncing.

To elucidate the mechanism underlying the interesting pancake bouncing phenomenon observed in our experiments, we acquired the drop spreading diameter *D* and the drop thickness *H* on the HMS substrate over time based on the photographic sequences in Fig. 3a,b. Figure 4a depicts the temporal evolution of *D* and *H* on the HMS substrate with and without the pancake bouncing phenomena. For clarity, we defined the spreading time corresponding to a local minimum drop thickness and a local maximum drop spreading diameter as 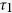 and 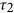 respectively. The drop spreading diameter and thickness at 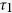 and 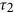 are designated as *D*_1_ and *H*_1_, and *D*_2_ and *H*_2,_ respectively. In the symmetric rebound, the occurrence of the minimum drop thickness was synchronous with the maximum spreading diameter at 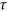 = 6.7 ms. Moreover, in most spreading processes, the increase in the drop spreading diameter was accompanied with a reduction of the drop thickness, suggesting a synchrony in the maximum spreading diameter and the minimum drop thickness.

However, in the case of pancake bouncing, the minimum drop thickness and the maximum spreading diameter emerged at 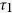 = 3.5 ms and 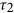 = 4.9 ms respectively, indicating the breakdown of time synchrony observed in the symmetric rebound. After reaching its minimum thickness at 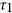 = 3.5 ms, the drop continued to spread with an increased spreading diameter until yielding a global maximum spreading at 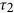 = 4.9 ms. Figure 4b plots the time evolution of *D*_2_/*D*_1_ and *H*_2_/*H*_1_ with and without pancake bouncing phenomena under different *We*. It is found that for the pancake bouncing, *D*_2_/*D*_1_ and *H*_2_/*H*_1_ are always larger than the unity whereas the ratios maintain a constant unity in the case of symmetric rebound. Moreover, temporally, in all the pancake bouncing experiments, we found that 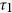 and 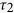 are comparable with each other, yet maintaining a relationship of 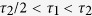. This means that the drop impact process involves a delicate time scale competition in the emergence of maximum spreading diameter and minimum drop thickness. Thus, the manifestation of non-synchrony as well as an intricate time competition in the emergence of minimum drop thickness and maximum spreading diameter might be essential temporal and spatial signatures of the pancake bouncing. Note that, there is no pancake bouncing until *We*_1_ = 14.2 in Fig. 4b, owing to insufficient energy storage. After the penetrated liquid reverses back, the upward kinetic energy is not large enough to lift the drop directly.

**Figure 5:**
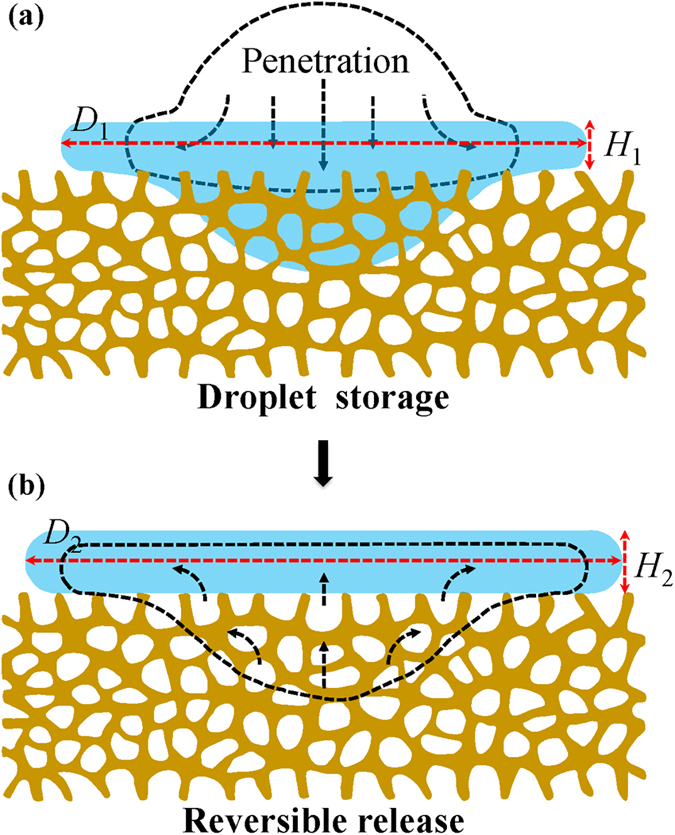
Sketch of liquid storage and release during the droplet impact. (**a**) Part of the liquid penetrating into the porous networks. (**b**) The penetrated liquid reverses back.

With these pictures in mind, we hypothesize that the pancake bouncing might involve a remarkable and additional motion in the vertical direction (as schematic in Fig. 5) during the earlier impact stage, which is unlike the impact on the traditional superhydrophobic surface on which the earlier stage liquid motion primarily takes place in the lateral direction. Liu *et al.* have shown that, during the drop impact on porous surface, part of the liquid would penetrate into the surface interspace in a localized region with the radius approximately equivalent to the initial drop radius^7^. The vertical drop motion will then lead to a significant liquid storage in the HMS substrate and subsequently the stored liquid extrudes out. Eventually the liquid is reversibly released back and the drop jumps like a pancake. Such a complex, transient, dynamic and muiti-scale process involves a delicate interplay between the drop impacting kinetics, surface morphology and surface chemistry, which might be endowed by a synergistic cooperation of micro/nanostructure of our HMS substrate at the spatial and temporal scales.

### Effect of surface morphology

To justify our hypothesis, we compared two resistance forces to which the drop was subjected to during drop motion along the vertical and lateral directions. In the vertical direction, the resistance force *P*_*n*_ (per unit area) against the drop penetration is scaled as *γ*/*d*_2_^27^. Similarly, the resistance force (per unit area) 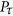 against the lateral drop spreading, which should be proportional to the pillar height *h*_1_ and the areal pillar density proportional to 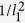, can be expressed as 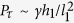 and hence 
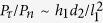
28. For our HMS substrate, 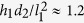. For almost all of the experiments reported in the literature^9^,^29^,^30^,^31^,^32^, this parameter takes values between 0.06 and 0.75, smaller than the threshold demonstrated in our work by almost one order of magnitude. On such surfaces, either the liquid penetration is insignificant, e.g., owing to too narrow and/or too short posts, or the capillary energy stored cannot be rectified into upward motion adequate to lift the drop, e.g., owing to an unwanted Cassie-to-Wenzel transition^31^,^33^,^34^,^35^,^36^. Thus, it is reasonable to argue that different from the bouncing on the conventional superhydrophobic surfaces, the lateral drop spreading and the vertical liquid penetration are both energetically preferred during the drop impact on the HMS substrate, though the depth of penetration is also dependent on the impinging velocity.

**Figure 6:**
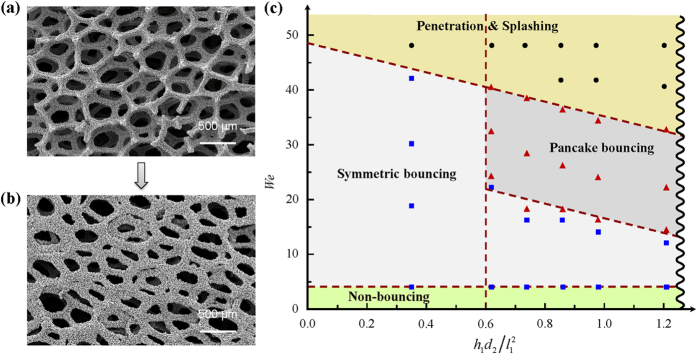
SEM images of the substrates and schematic of the phase diagram. (**a**) SEM image of the original substrate. (**b**) SEM image of the substrate without pillars by polishing. (**c**) Schematic of the phase diagram illustrating three impact regimes of drop impact in terms of 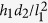. Blue squares: symmetric bouncing; Red up triangles: pancake bouncing; Black dots: penetration & splashing.

In order to verify the matching of 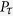 and *P*_*n*_ for pancake bouncing, we deliberately decreased the thickness of the substrates, which means the decrease of pillar height (*h*_1_) and/or pore size (*d*_2_). Figure 6a,b show the SEM images of the substrates with and without pillars, respectively. On the surface without pillars, pancake bouncing disappears regardless of the impact velocities. As shown in the schematic of the phase diagram in Fig. 6c, there are three impact regimes of drop impact in terms of 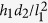. They are symmetric bouncing, pancake bouncing, and penetration or splashing, which are highly coupled depending on the interplay between the substrate morphology and the impacting kinetics. The critical 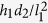 separates the first two regimes is ~0.6. In the first regime, the lateral spreading is energetically favored over the vertical penetration due to the smaller 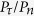 similar to that on conventional superhydrophobic surfaces. Accordingly, the particular pancake bouncing can not be observed, which is well confirmed by our experimental observations on the substrates with small 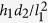. In the second regime, since 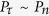, the lateral spreading and the vertical penetration are both favored. The pancake bouncing occurs over a relatively large Weber number range. Finally, if the drop comes to the substrate with a high impact velocity, large propotion of the drop would penetrate into the porous matrix, splash occurs and pancake boucing disappeared. The availability of the phase diagram will also allow us to predict any impact dynamics senario.

In conclusion, we report a pancake-bouncing phenomenon on a facilely fabricated superhydrophobic copper foam that features with two-tier roughness and multilayered porous structure. Using the experimental and theoretical analyses, our study reveals that a matching between the lateral and vertical liquid motion is necessary to allow for a sufficient drop deformation and energy storage as well as effective energy release to engender a vertical pancake bouncing. Compared to the conventional rebounding, the shortened contact time endowed by the pancake bouncing is constructive for a wide range of applications where fast drop departure is preferred. Additionally, engineering superhydrophobic surface with enhanced stability against the Cassie-to-Wenzel transition by the external perturbation such as vibration^37^,^38^, condensation^39^,^40^ and electrowetting has proved challenging in the past decade, the hierarchical surface with open-channel as well as proper morphology developed here might open a new avenue to develop robust superhydrophobic surfaces that overcome serious limitations confronted by the conventional superhydrophobic materials.

## Additional Information

**How to cite this article**: Liu, Y. and Wang, Z. Superhydrophobic porous networks for enhanced droplet shedding. *Sci. Rep.* **6**, 33817; doi: 10.1038/srep33817 (2016).
